# Contribution of the immune bone marrow microenvironment to tumor growth and bone deconstruction: implications for improving immunotherapeutic strategies in bone metastasis

**DOI:** 10.1016/j.neo.2025.101224

**Published:** 2025-09-02

**Authors:** E. Massy, C.B. Confavreux, M. Point, E. Bonnelye, P. Clézardin

**Affiliations:** a Service de Rhumatologie Sud, Centre Expert des Métastases Osseuses (CEMOS), Institut de Cancérologie des Hospices Civils de Lyon, Pierre Bénite, France; b INSERM, UMR 1033, LYOS, Lyon, France; c Université Claude Bernard Lyon I, Villeurbanne, France; d Centre de Recherche en Cancerologie de Lyon (CRCL) - UMR INSERM 1052 CNRS 5286, Lyon, France; e Université Lille, CNRS, INSERM, CHU Lille, UMR9020-UMR1277-CANTHER- Cancer Heterogeneity, Plasticity and Resistance to Therapies, Lille, France; f Division of Clinical Medicine, School of Medicine and Population Health, University of Sheffield, Sheffield, UK

**Keywords:** Bone metastasis, Immunity, Micro-environment, Immunotherapy, Bone resorptive agents

## Abstract

Bone metastases are frequent complications of many solid tumors, leading to painful skeletal morbidities and increasing mortality for patients with advanced cancer. Once in bone, cancer cells deregulate bone homeostasis, altering the functions of bone-forming (osteoblasts) and bone-resorbing (osteoclasts) cells, which results in skeletal deconstruction. Aside from bone cells, cancer cells in the bone marrow interact with other cell populations, including immune cells that also play an integral part in the regulation of bone homeostasis. In this respect, immune checkpoint inhibitors (ICIs) have become a standard of care in immunotherapy for the treatment of patients with advanced cancer. Strikingly, however, those with bone metastases have a shorter survival when treated with ICIs than ICI-treated cancer patients without bone metastases. In this Review, after presenting the immune cells involved in bone metastasis, we review preclinical and clinical findings assessing ICI efficacy both in bone and extraosseous metastases, and we discuss the clinical utility of using bone-targeted agents —including denosumab and bisphosphonates— to improve anti-tumoral efficacy of ICI treatments in patients with cancer and bone metastases.

## Introduction

Bone metastases are frequent complications of many solid cancers of which prostate, breast and lung cancers are the most frequent [1]. Bone metastasis development first requires cancer cell extravasation and homing to the bone marrow through interactions with endothelial cells and osteoblasts in order to develop a bone metastatic niche [1,2]. Once in bone, cancer cells disrupt bone homeostasis, promoting bone resorption by osteoclasts and altering bone formation by osteoblasts [1]. In turn, growth factors released from the bone matrix promote skeletal tumor growth, creating a vicious cycle that leads to the formation of osteolytic or osteoblastic/mixed lesions [1]. In the past years, single-cell and spatial technologies have revolutionized our understanding how cancer cells and non-cancer cells interact in complex ecosystems that shape disease progression both in primary tumor and distant metastasis [3,4]. Specifically, single-cell profiling of bone metastasis from different carcinomas (*e.g*. breast, lung, prostate, kidney) revealed that, aside from bone cells, cancer cells in the bone marrow interact with multiple cell types, the dominant cell type being immune cells [5]. This may be not surprising, considering that the bone marrow is a microenvironment where immature immune cells develop [6]. In bone metastases, three distinct immune ecosystems have been characterized with an enrichment of macrophages/osteoclasts, regulatory/exhausted T cells, or monocytes [5]. These immune ecosystems do not stem from matched primary tumors, but represent divergent evolution propelled by different bone-derived sources of selective pressure, such as confinement to mineralized bone tissue and immunosurveillance [4,5]. Furthermore, an enrichment of regulatory/exhausted T cells is associated with a poor relapse-free survival of patients with breast cancer and bone metastases [5], suggesting that these distinctive immune ecosystems could be prognostic of clinical outcomes and indicative of treatment response. Indeed, immune checkpoint inhibitors (ICIs) are indicated immunotherapies in various solid cancers (lung, prostate and renal cell carcinoma, melanoma), which have a high propensity to metastasize to bone [7,8]. Strikingly, however, patients with cancer and bone metastases have a poorer survival in response to ICIs than cancer patients without bone metastases [8]. There is therefore a need to understand the reasons for this dismal effect of ICI therapy in patients with cancer and bone metastases.

Herein, after presenting the immune cells involved in bone metastasis formation and providing a compendium of clinical studies assessing ICIs in bone and extraosseous metastatic disease, we discuss the clinical utility of using bone-targeted agents in combination with ICIs to improve anti-tumoral efficacy of treatments in patients with cancer and bone metastases.

## Immune cells and bone metastasis

**Fig. 1:**
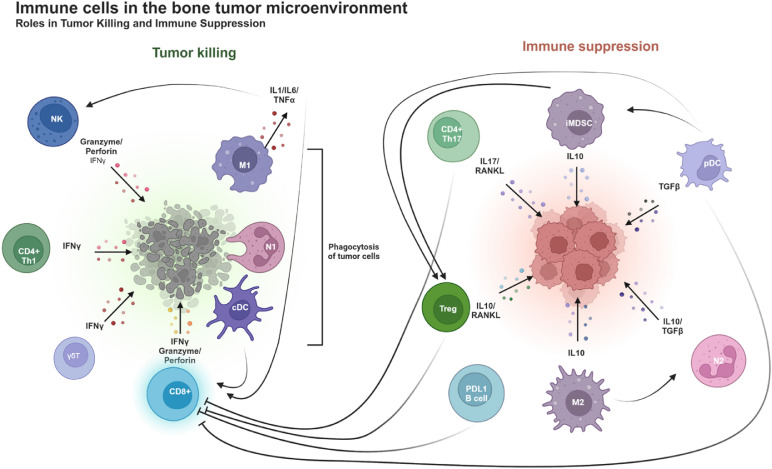
Distinct roles of immune cells in the bone tumor microenvironment. **Left-hand panel:** several immune cells work to destroy tumor cells: NK cells release granzyme and perforin, which directly induce tumor cell lysis. CD4+ Th1, γδ T and CD8+ T cells secrete IFNγ, a cytokine that enhances the immune response by activating other immune cells and promoting tumor cell death. CD8+ T cells also produce granzyme and perforin, mediating direct cytotoxicity. M1 macrophages produce pro-inflammatory cytokines like IL-1, IL-6, and TNFα, which support inflammation and anti-tumor responses, and they also phagocyte tumor cells. N1 neutrophils and conventional dendritic cells (cDCs) contribute to the direct tumor cells elimination through immune activation. **Right-hand panel:** specific immune cells promote tumor growth by suppressing the anti-tumor immune response. M2 macrophages and immature myeloid-derived suppressor cells (iMDSCs) secrete IL-10, an immunosuppressive cytokine that dampens anti-tumor immune responses, allowing tumor cells to evade immune surveillance. Tregs produce IL-10, which inhibits the activity of other immune cells. Plasmacytoid dendritic cells (pDCs) release TGFβ, a cytokine that suppresses immune responses and promotes tumor growth. N2 neutrophils further contribute to immune suppression by supporting a pro-tumoral environment. Overall, this figure illustrates the dynamic balance between immune cells that actively fight tumor cells and those that create an immune suppressive microenvironment, enabling tumor cells growth.

The activation of the innate immune system and the coordination between innate and adaptive immune cells are critical to launch efficient and effective immune responses against tumor progression [9], as illustrated in Fig. 1 for bone metastasis.

### Innate immune cells

Innate immune cells [myeloid-derived suppressor cells, macrophages, neutrophils, dendritic cells, natural killer and gamma-delta T lymphocytes] contribute to bone metastasis formation, harboring tumor-promoting or tumor-suppressing activities (**Supplementary Table S1**).

#### Myeloid-Derived suppressor cells

Myeloid-Derived Suppressor Cells (MDSCs) represent one third of bone marrow immune cells [9]. MDSCs suppress the activation of macrophages and the antigen-presenting ability of dendritic cells (DC); they also suppress the cytotoxicity of natural killer (NK) cells and enhance T regulatory (Treg) cell expansion [10]. In preclinical models of breast cancer and multiple myeloma, MDSCs expand during skeletal tumor progression and promote tumor-induced bone destruction [11].

#### Macrophages

Macrophages can polarize into two different subtypes, M1 and M2, the latter being regarded as tumor-associated macrophages (TAMs) [9]. M1 macrophages secrete pro-inflammatory cytokines [IL1, IL6, and tumor necrosis factor (TNF)-α] that activate cytotoxic T lymphocytes (CD8+ T cells) and NK cells to eliminate cancer cells [9]. M1 macrophages also participate to the clearance of apoptotic cancer cells (a process called efferocytosis). However, as exemplified in a prostate cancer model, efferocytosis of apoptotic cancer cells by bone-marrow derived macrophages can induce the expression of pro-inflammatory chemokines such as C-X-C motif ligand 5 (CXCL-5) that accelerates skeletal tumor growth [12]. Indeed, a number of tumor-derived chemokines [CXCL-5, CXCL-12, C-C motif ligand 2 (CCL-2), macrophage colony-stimulating factor (M-CSF)] in prostate or breast cancer promote M2 macrophage polarization and drive TAM recruitment to bone [13,14]. In turn, TAMs secrete high levels of IL10 and transforming-growth factor (TGF)-ß that decrease the activation of CD4+ and CD8+ T cells during tumor progression and bone metastasis formation [14]. These experimental findings are in line with the observation that M2 macrophages in bone metastasis specimens from patients with breast or prostate cancer are increased compared to matched primary tumors or lymph node metastases [5,14]. Thus, TAMs provide an immunosuppressive environment that sustains tumor growth in bone.

#### Neutrophils

Similar to macrophages, neutrophils within tumors can be divided between anti-tumor N1 neutrophils (CD66b^+^ CD11b^+^ CD170^low^) that restrain tumor growth at early stages of the disease and pro-tumor N2 neutrophils (CD66b^+^ CD11b^+^ CD170^high^ PDL1^+^) that tend to promote tumor growth and metastasis [15]. In prostate cancer models of bone metastasis, bone marrow neutrophils induce apoptosis of tumor cells*,* reducing skeletal tumor growth [16]. However, as bone metastases in animals progress over time, neutrophils from late-stage skeletal tumors lose their cytotoxic potential against prostate cancer cells [16], recapitulating the N1 to N2 transition of neutrophils during cancer progression [9]. These findings [16] likely explain why a high neutrophil-to-lymphocyte ratio in patients with cancer and bone metastasis is associated with a poor overall survival [17]. Moreover, an abnormal and rapid increase of immature neutrophils in mice has been observed in the bone tumor microenvironment during the progression of bone metastases, accounting for up to 80 % of total neutrophils at advanced stages [18]. Indeed, immature neutrophils (CD66b⁺CD11b⁺CD49d⁻CD10⁻) have immunosuppressive activity; they inhibit CD8⁺ T cell activation and alter IFN-γ production [18].

#### Dendritic cells

DCs are specialized antigen-presenting cells that derived from hematopoietic bone marrow progenitor cells; they differentiate into two main cell populations: myeloid (or conventional) DCs (cDCs) characterized by XCR1, CD370, CD141 expression and plasmacytoid DCs (pDCs) characterized by CD303, CD123, CD304 expression [19]. Tumor antigen-pulsed cDCs are able of inducing activation of CD8+ T cells to mediate anti-tumor responses [9]. This could explain why a high number of cDCs in primary tumors from patients with early invasive breast cancer (n = 110) is associated with a lower risk of bone relapse (*P* = 0.01), and a longer overall survival (*P* = 0.02) [20]. cDCs are also present in bone metastatic biopsies from patients with different cancer types, as opposed to that observed in normal bone marrow biopsies [5]. However, the role of cDCs in experimental models of bone metastasis has never been explored. In contrast, following depletion of pDCs in immunocompetent syngeneic breast cancer models, the activity of CD8+ T cells in the bone marrow increases, which leads to a reduction of bone metastases in animals [21]. This suggests that pDCs are critical regulators of bone metastasis, but no conclusion can be drawn at this point due to the paucity of informative clinical studies in the literature.

#### Natural killer cells

Natural killer (NK) cells play a key role in tumor immunosurveillance and limitation of metastasis in mice and human [9]. For example, the impairment of NK cell-mediated antitumor immunity with a JAK/STAT inhibitor enhances skeletal tumor burden in animal models of breast cancer metastasis [22]. Indeed, activated NK cells release cytolytic granules (granzymes and perforin) and interferon (IFN)-γ that cause the elimination of tumor cells [9]. Pathways of IFN induction are regulated by IFN regulatory factors (IRF3, IRF5 and IRF7) [9,22]. Bidwell and colleagues [23] found that *irf7* expression was suppressed in bone metastatic 4T1.2 breast cancer cells, whereas its forced expression in 4T1.2 cells restored an antimetastatic immune response in immunocompetent tumor-bearing animals [23]. Furthermore, NK cells regulate tumor cell dormancy [24], which is the result of a balance between cancer stem cell (CSC) proliferation and quiescence [1]. In breast cancer models, NK cells mediate lysis of proliferative CSCs *in vivo*, compared with quiescent CSCs [24]. Overall, these findings [[22], [23], [24]] indicate that NK cells contribute to the reduction of bone metastasis formation.

#### Gamma delta T cells

Human gamma delta (γδ) T cells constitute a small proportion (1-5 %) of the lymphocytes that circulate in blood [25], and among these γδ T cells, there is a subset called Vγ9Vδ2 T cells that is enriched in many peripheral tissues (skin, intestines, and lungs), involved in the regulation of bone physiology [25], and having significant antitumor activity [26]. Contrary to the T cell receptor (TCR) chains αβ that recognize antigens bound to major histocompatibility complex (MHC) molecules, γδ-TCRs directly recognize these antigens, which place γδ T cells at the border between innate and adaptive immunity [25,26]. In this respect, Vγ9Vδ2 T cells react within hours to phosphorylated metabolites [*e.g*., isopentenyl pyrophosphate (IPP)] of the mevalonate pathway in eukaryotic cells [26]. In a preclinical model of human breast cancer, bisphosphonate treatment (zoledronate or risedronate) of immunodeficient animals promotes the accumulation of human Vγ9Vδ2 T cells in tumor xenografts producing high IPP levels, which leads to tumor regression [27,28]. Similarly, in a preclinical murine model of castration-resistant prostate cancer, zoledronate enhances the rate of regression of intratibial prostate tumors induced by γδ-T cells [29].

### Adaptive immune cells

Approximately 8-20 % of mononuclear cells in the bone marrow are lymphocytes, with a T:B cell ratio of 5 to 1 [30]. Among αβ T cells in the bone marrow, there are CD8+ and CD4+ T cells which have cytotoxic and auxiliary regulatory functions, respectively [9,31]

#### CD8+ T cells

CD8+ T cells can be divided into different subsets including naïve, effector, memory, and exhausted T cells [9]. In bone metastasis biopsies from patients with different cancer types, CD8+ effector T cells are almost always found, and 50 % of these biopsies also contain CD8+ exhausted T cells, whereas only CD8+ naïve T cells are present in normal bone marrow biopsies [5]. In mouse models, activated CD8+ T cells reduce bone metastasis formation, whereas the deprivation of these T cells increases skeletal tumor growth, demonstrating that they are critical regulators of bone metastasis formation [32]. Nevertheless, as bone metastases progress and cancer-induced bone destruction occurs, TGFβ released from resorbed bone inhibits T cell-mediated anti-tumor immune responses [33]. MDSCs also expand during skeletal tumor progression and inhibit CD8+ T cell functions [11]. Furthermore, Dickkopf-1 (DKK-1), an antagonist of Wnt (Wingless/int) proteins that is secreted from tumor cells (breast, prostate, myeloma), induces an immature-like functional state in neutrophils [18]. In turn, immature neutrophils secrete CHI3L3 (chitinase-3-like protein 3), which directly inhibits the anti-tumor response of CD8 + T cells [18]. Taken together, these experimental findings [11,18,33] likely explain why the fate of CD8+ effector T cells can be heavily biased toward exhaustion in human bone metastases [5].

#### CD4+ T cells

Influenced by the different cytokines present in the microenvironment, CD4+ T cells differentiate into T helper 1 (Th1), Th2, Th17, or Treg subsets [9].

Tregs are frequently increased in bone metastases from patients with cancer (especially kidney, but also lung, breast, or prostate), when compared to that observed in the bone marrow from healthy individuals [5]. This enrichment of Tregs is also associated with a poor relapse-free survival of patients with breast cancer and bone metastases [5]. A study of Tregs in mammary carcinoma models demonstrated that tumor-infiltrating Tregs are a major source of receptor activator of nuclear factor-κB (RANK) ligand (RANKL), which stimulates tumor progression and lung metastasis from RANK-expressing mammary carcinoma cells [34]. This could also have implications for RANK-expressing bone metastasis where the same mechanisms are likely to be at play [35]. For example, mice lacking soluble RANKL (*Tnfsf11 ^ΔS/ΔS^*) have normal bone homeostasis and develop a normal immune system, but they display a marked reduced formation of bone metastases after inoculation of RANK-expressing melanoma or breast cancer cells, indicating that bone-derived soluble RANKL attract RANK-expressing tumor cells in the bone marrow [36]. In bone, RANKL is produced by different bone cell types (osteoblasts, osteocytes) and its binding to RANK, a transmembrane receptor expressed by osteoclasts precursors, induces (in the presence of M-CSF) the differentiation of osteoclasts precursors into mature osteoclasts, which then resorb the mineralized bone matrix [1,6]. RANKL is also expressed by B and T cells (including Tregs) but does not significantly contribute to the physiological regulation of osteoclast differentiation [6]. Moreover, activated Tregs produce factors (IL-4, IL-10 and IFN-γ) that can potentially inhibit osteoclast differentiation [1,6]. In this respect, the intravenous injection of activated Tregs to immunodeficient NOD/SCID mice bearing human PC3 prostate cancer skeletal lesions leads to a reduction of bone destruction, due to the osteoclast-inhibitory effect of Treg cells [37].

As opposed to Tregs, Th17 cells produce IL-17, which stimulates osteoclast differentiation by increasing RANKL expression on osteoclast-supporting mesenchymal cells, thereby contributing to bone destruction under pathological conditions such as rheumatoid arthritis and cancer bone metastasis [1]. In this respect, the knock down of RANKL expression in Th17 cells significantly reduces the formation of osteolytic bone lesions in a 4T1 breast cancer model [38].

Th1 cells reduce cancer-induced bone destruction. This is explained by the fact that Th1 cells kill tumor cells and inhibit osteoclast formation *via* the release of IFN-γ [39]. However, high amounts of TGF-β are released from resorbed bone during bone metastasis formation and, as observed in an animal model of prostate cancer, TGF-β restrains Th1 cell development and promote Th17 cell expansion in the bone marrow [40]. Conversely, anti-TGF-β treatment of animals bearing prostate cancer bone metastases allows Th1 development in the tumor microenvironment [40].

Th2 cells inhibit osteoclast formation by producing IL-4 [6], but their role in bone metastasis formation is unknown.

Collectively, these findings [[37], [38], [39], [40]] indicate that specific subsets of CD4+ T cells help create a sanctuary in the bone marrow that protects tumor cells from immune attack. Not only Tregs impair CD8+T cell proliferation, but there is also a balance between Tregs and Th17 cells that regulates cancer-induced bone destruction [37,40]. Furthermore, TGF-β released from resorbed bone promotes Th17 cell expansion at the expense of the Th1 cell lineage, thereby preventing immune elimination of tumor cells in the bone marrow [40].

#### B cells

The existence of B cells in the tumor microenvironment has repeatedly been reported in solid cancer [41], B cells preferentially localize to structure (tertiary lymphoid structures or TLS) that are made up of a predominant B cell-germinal center surrounded by an immune cell aggregate consisting of T cells, DCs, MDSCs, and NK cells [41]. The formation of these TLS within the tumor microenvironment is correlated with anti-tumor control [41].

In melanoma, B cells express significantly higher programmed cell death-1 (PD-1) ligand 1 (PD-L1) levels in bone metastases than in primary tumors, and PD-L1-positive B cells suppress T cell response, thereby creating an immunosuppressive environment in the bone marrow [42]. The functions of B cells (and TLS) in bone metastasis formation are currently unknown.

## Perspectives of immunotherapy for the treatment of bone metastasis

Immune checkpoint inhibitors (ICIs) have revolutionized the treatment of advanced cancers [7]. Antibodies targeting checkpoint molecules such as cytotoxic T-lymphocyte associated-protein 4 (CTLA-4), PD-1, and PD-L1 have proven effective as monotherapies in several malignancies—including melanoma, non-small cell lung cancer (NSCLC), renal, urothelial and head and neck squamous cell carcinoma—by reversing immune tolerance and promoting T cell activation [[43], [44], [45], [46], [47]]. For instance, pembrolizumab (an anti-PD1 antibody) significantly improves overall survival (OS) in NSCLC (22 *vs* 10.6 months; p < 0.0001) [45], head and neck squamous cell carcinoma (13 *vs* 10.7 months; p = 0.0067) [47], and urothelial carcinoma (10.3 *vs* 7.4 months; p = 0.004) [44]. Responders often display an increased number of circulating CD8+ T cells [7]. Moreover, combining CTLA-4 and PD-1/PD-L1 blockade enhances therapeutic outcomes due to their complementary mechanisms—CTLA-4 inhibition boosts T cell priming, while PD-1/PD-L1 blockade reactivates exhausted CD8+ T cells [7].

Despite these advances, there are preclinical and clinical evidence that patients with bone metastases show diminished responses to ICIs compared to those without bone involvement [8]. Liver and lung metastases can also exhibit different responses to immunotherapies [48]. Herein, we review preclinical and clinical findings assessing ICI efficacy in bone and extraosseous metastatic sites, and we discuss the clinical utility of using bone-targeted agents —including denosumab and bisphosphonates— to improve anti-tumoral efficacy of ICI treatments in patients with cancer and bone metastases.

### ICIs in metastatic bone disease

The bone tumor microenvironment is considered as "immune-cold" because cytotoxic T cells are blunted by different immunosuppressive cells, such as MDSCs, TAMs, immunosuppressive neutrophils, Tregs and Th17 cells [9,11,18], which may likely cause a diminished response to ICI therapies. For example, as previously discussed in *section 2.2.2*, TGF-β released from resorbed bone during bone metastasis formation promotes Th17 cell expansion at the expense of the cytotoxic Th1 cell lineage, thereby preventing immune elimination of tumor cells in the bone marrow [40]. Moreover, DKK1, which is highly expressed in bone metastases, induces an immature-like functional state in neutrophils via expression of CHI3L3, which directly inhibits the cytotoxic activity CD8+ T cells in the bone marrow [18]. Another reason for the diminished response to immunotherapy may be the ICI treatment itself which, by enhancing immune activation in the bone marrow, can result in osteoclast activation, bone loss and fracture, as previously reported in preclinical models [49] Indeed, owing to their growing use in clinical oncology, ICIs can cause a wide range of immune-related adverse events in various organs, including rheumatologic toxicities such as inflammatory arthritis, bone loss, and osteoporosis [50,51]. For example, in patients with melanoma treated with ICI, there is almost a 2-fold increase in the incidence rate of osteoporotic fractures over the first year after ICI initiation [51]. The presence of oncogenic driver mutations within NSCLC tumors is also a strong determinant of ICI response. *EGFR* mutations correlate with reduced PD-L1 expression and immune evasion [52], and pembrolizumab (an anti-PD1 antibody) yields poor responses in *EGFR*-mutant tumors, even with high PD-L1 levels [52,53]. *KRAS/TP53* co-mutations respond favorably to ICIs [objective response rate (ORR): 35.7 %], while *KRAS/STK11* co-mutations respond poorly (ORR: 7.4 %) [53,54]. Similarly, NSCLC patients with *ALK* rearrangements respond poorly to ICIs, with ORRs between 0–3 % [55]. Thus, oncogenic driver mutation types in NSCLC could also have an impact on the response to ICI therapy of patients with bone metastasis.

Clinical studies assessing ICI efficacy in metastatic bone disease remain limited, but suggest worse outcomes at least for patients with NSCLC, renal cell carcinoma, castration-resistant prostate cancer (CRPC), or urothelial cancer (Table 1). Specifically, in NSCLC, retrospective studies show shorter PFS and OS for patients with bone metastases treated with anti-PD1 agents (pembrolizumab or nivolumab, another anti-PD1 antibody) compared to those without [[56], [57], [58], [59], [60], [61], [62]]. Combination therapies with chemotherapy may overcome this negative effect, as outcomes were similar regardless of bone involvement [61,62]. One study found no PFS difference in pembrolizumab-treated patients with high PD-L1 expression (TPS ≥ 50 %), highlighting the importance of stratification [59]. In chemotherapy-naive patients with metastatic CRPC and no known visceral metastases (brain, lung, and liver metastases), ipilimumab (an anti-CTLA4 antibody) failed to improve OS, compared to placebo, but modestly extended PFS [63,64]. Similarly, pembrolizumab showed limited antitumor activity in a subset of bone-predominant CRPC patients [65]. Trials combining ICIs with radium-223 or radiotherapy yielded inconclusive benefits, though one study noted higher long-term OS with ipilimumab post-radiotherapy [66]. In breast cancer, ICIs have recently been approved for PD-L1–positive metastatic triple-negative breast cancer, improving both PFS and OS [[67], [68], [69]]. Although over 20 % of participants had bone metastases, no subgroup analysis was performed. Ongoing trials aim to address this gap (NCI-2016-02057).

**Table 1:** Clinical trials involving patients with cancer and bone metastasis treated with immune checkpoint inhibitors.

Clinical trials involving patients with cancer and bone metastasis treated with ICIs (*)								
Cancer type	ICI Treatment (⁎⁎)	Study type	Total number of patients (n)	Without BM (n)	With BM (n)	Clinical outcomes (with versus without BM)	P value	Reference
**NSCLC**	Nivolumab	retrospective	1588	962	626	PFS; 3.0 vs 4.0 months OS; 7.4 vs 15.3 months	<0.0001 <0.0001	Landi, 2019[53]
	Pembrolizumab, Nivolumab or Atezolizumab + Chemotherapy	retrospective	101	68	33	PFS; 10.1 vs. 12.1 months OS; 24.6 months vs. NR	NS NS	Li, 2020[58]
	Pembrolizumab, Nivolumab or Atezolizumab		103	69	34	PFS; 4.2 vs. 6.7 months OS; 12.5 vs. 23.9 months	0.048 0.0036	Li, 2020[58]
	Pembrolizumab, Nivolumab or Atezolizumab	retrospective	330	206	124	OS; 5.9 vs 13.4 months	<0.001	Qin, 2021[55]
	Pembrolizumab	retrospective	213	154	59	PFS; 7.7 vs 8.7 months	0.986	Kawachi, 2020[56]
	Pembrolizumab	retrospective	1126	627	272	PFS; 4.5 vs 11 months OS; 10.9 vs 27.5 months	<0.0001 <0.0001	Cortellini, 2020[54]
	Pembrolizumab, Nivolumab or Atezolizumab	retrospective	39	29	10	shorter PFS in patients with BM [HR= 2.74 (95CI% 1.13-6.66)]	0.026	Dall'Olio, 2021[57]
	Pembrolizumab, Nivolumab, Sentilimab, Camrelizumab, Toripalimab or Tislelizumab + Chemotherapy	retrospective	99	51	48	OS; 14.2 months vs NR	NS	Zhu, 2022[59]
	Pembrolizumab, Nivolumab, Sintilimab, Camrelizumab, Toripalimab or Tislelizumab		38	27	11	OS; 3.3 vs 23.4 months	0.0004	Zhu, 2022[59]
**RCC**	Nivolumab (n=76) vs everolimus (n=70) in second-line therapy of bone metastatic patients after failure of antiangiogenic therapy	prospective	146		146	OS; 18.5 vs 13.8 months	0.24	Escudier, 2017
	Nivolumab	retrospective	68	47	21	ORR; with BM (5%) compared to lung (36%) and liver metastasis (50%)	0.017	Negishi, 2021
	Nivolumab in second-line therapy after failure of antiangiogenic therapy	prospective	720	526	194	OS; 17.9 vs 26.1 months PFS; 2.8 vs 4.6 months	0.0707(#) 0.0045	Velev, 2023
	Nivolumab + Ipilimumab	retrospective	36	27	9	12-month OS rate; 41.7% vs 82.7%	0.021	Pham, 2022
**CRPC**	Ipilimumab (n=399) vs placebo (n=199) in the first line treatment of chemotherapy-naïve patients without visceral metastases	prospective	598	124	471	OS; HR=1.19 (95% CI 0.88-1.52) vs 0.80 (95%CI 0.50-1.28)	NS	Beer, 2017[60]
	Ipilimumab (n=399) vs placebo (n=400) after bone-directed radiotherapy in metastatic patients who had received chemotherapy previously	prospective	799	0	799	5-year OS rate in the ipilimumab treated arm 3-fold higher than in the placebo treated BM arm (7.9% vs 2.7%)	NR	Fizazi, 2020[61]
	Pembrolizumab in metastatic patients who had received chemotherapy and targeted endocrine therapy previously	prospective	258	199	59	OS; 14.1 months (95% CI 10.8-17.6) vs 8.1 months (95%CI 6.6-10.7)	NR	Antonarakis, 2020[62]
	Atezolizumab + radium-223 (n=17) vs radium-223 (n=13) in bone metastatic patients who had received endocrine therapy previously	prospective	30	0	30	OS; 16.3 vs 15.9 months	NR	Fong, 2021[63]
	Pembrolizumab + radium-223 (n=29) vs radium-223 (n=13) in bone metastatic patients who had received chemotherapy and endocrine therapy previously	prospective	42	0	42	OS; 16.9 vs 16.0 months	NR	Choudhury, 2024
**Urothelial**	Pembrolizumab	retrospective	69	62	7	ORR; 31.3% for lung, 29.0% for lymph node, 23.1% for liver and 28.6% for BM	0.616	Furibayashi, 2021
	Pembrolizumab	retrospective	136	111	25	ORR; 7% (BM) vs 30% (other metastases)	0.0053	Shimizu, 2022

(*) BM = bone metastasis; CRPC = castration-resistant prostate cancer; HR = hazard ratio; ICI = immune checkpoint inhibitor; NA = not applicable; NR = not reached; NS = not significant; NSCLC = non-small cell lung cancer; OS = overall survival; PFS = progression free survival, ORR = objective response rate, RCC = renal cell carcinoma.

(**) ICIs: anti-PD-1 (Camrelizumab, Pembrolizumab, Nivolumab, Sintilimab, Tislelizumab, Toripalimab), anti-PDL-1 (Atezolizumab), and anti-CTLA-4 (Ipilimumab) checkpoint inhibitors.

(#) corrected p value.

### ICIs in extraosseous metastatic disease

Liver metastases correlate with diminished immunotherapy efficacy in a wide variety of human cancers (melanoma, NSCLC, urothelial and renal cancers) [48]. In animal models of melanoma, liver metastases (but not subcutaneous tumors or lung metastases) recruit circulating monocytes and polarize them into M2 macrophages [48]. In turn, circulating Fas-expressing CD8+ effector T cells undergo apoptosis in the liver following their interaction with FasL-expressing M2 macrophages [48]. Consequently, liver metastases deplete peripheral CD8+ T cells, causing systemic immunotherapy resistance. Of note, there was no mention of bone metastases in these studies, but liver, brain and lung metastases [48]. Nevertheless, as previously discussed (*section 2.1.2)*, M2 macrophages in bone metastases could also provide an immunosuppressive environment, contributing to local immunotherapy resistance. Having said that, some studies report shorter OS and reduced immunotherapy benefit in patients with NSCLC and liver involvement at baseline with a 5-year multivariate analysis confirming liver metastases as an independent predictor of increased risk of death following nivolumab treatment [70]. Strikingly, however, combining ICI therapy (atezolizumab, an anti-PD-L1 antibody) with chemotherapy and anti-angiogenic agents (bevacizumab) as first line treatment for patients with NSCLC and metastasis significantly improves PFS and OS, compared to chemotherapy + bevacizumab alone (7.4 *vs* 4.9 months) [71]. Both chemotherapy and bevacizumab may augment the antitumor efficacy of ICI through several mechanisms, including upregulating PD-L1 expression on cancer cells, explaining this benefit with respect to PFS [71].

Patients with melanoma and lung only metastases have better OS at 1 year (89 %) and ORR (62 %) to ICI therapy (pembrolizumab), compared to melanoma patients with liver metastases only (OS, 53 %; ORR, 22 %) [72]. A few additional clinical studies reported similar good responses to anti-PD1-based immunotherapies in patients with cancer (melanoma, hepatocellular and colorectal carcinoma) and lung metastases only [72]. Strikingly, the presence of bone metastases in patients with cancer and lung metastases (or with any other type of metastasis) diminishes ORR to ICI therapies at these extraosseous metastatic sites [73]. Using mouse models of cancer (melanoma, NSCLC, colon) and metastasis, it has been shown that osteolytic lesions remotely alter the immune environment at extraosseous metastatic sites [73]. Mechanistically, osteoclast-derived osteopontin (an extracellular matrix protein released during bone resorption) is driving resistance to ICI therapy by impairing CD8+ T cell recruitment and differentiation at these extraosseous metastatic sites [73]. Moreover, in animal models of breast cancer, host-derived osteopontin promotes the expansion of MDSCs at the lung metastatic site, further creating an immunosuppressive microenvironment [74].

### Combination therapy with ICIs and bone-targeted agents

Bone-targeted agents, such as bisphosphonates and denosumab, have become established in the systemic treatment of bone metastases to prevent the occurrence of skeletal-related complications associated with cancer-induced bone destruction [1,75]. Bisphosphonates inhibit osteoclast activity and reduce bone resorption [1,75]. Denosumab is a fully human, synthetic antibody that binds to RANKL with high affinity, preventing its interaction with RANK, thereby inhibiting osteoclast formation and, subsequently, bone resorption [1,75]. Denosumab has been shown to be superior in advanced breast and prostate cancer and non-inferior in other advanced solid cancers and myeloma to the bisphosphonate zoledronate for the prevention of skeletal-related complications [1,75]. Furthermore, RANK/RANKL are also expressed by various cancer and immune cells [1,76], suggesting the association of denosumab with ICIs would be the best therapeutic approach to improve ICI efficacy in metastatic bone disease.

Experimentally, in prostate cancer models with bone metastases, an anti-RANKL treatment enhances the efficacy of CTLA-4 and PD-1 blockade by reducing osteoclast-mediated release of TGF-β, which would otherwise suppress Th1 cell response and promote Th17 differentiation [40]. Anti-RANKL therapy also increases CD8+ T cell infiltration and reduces osteopontin production in mice bearing subcutaneous tumors (colon carcinoma, melanoma) and bone lesions, thereby improving the extraosseous anti-tumor effects of a PD-L1 inhibitor [73]. In contrast to anti-RANKL, zoledronate is unable to improve the extraosseous response to anti-PD-L1 treatment in mice with subcutaneous colon tumors (MC8) and bone lesions [73]. However, in a mouse model of breast cancer (4T1), a combined treatment with anti-PD-L1 and zoledronate does reduce subcutaneous tumor growth, compared to single therapies [77]. Using a syngeneic mouse model of breast cancer and bone metastasis, dual blockade therapy with anti-DKK1 and anti-PD-1 antibodies almost completely eliminates bone metastasis formation in animals, suggesting that DKK-1 blockade could be another therapeutic strategy to overcome ICI resistance in bone metastases [18].

Clinically, a few trials provide some evidence that combining ICI therapy with denosumab (or zoledronate) could improve tumor response to ICI therapy in patients with cancer and bone metastases (Table 2). In metastatic melanoma, patients receiving ICI + denosumab have improved OS compared to ICI alone, though differences are not statistically significant due to the small cohort size [[78], [79], [80], [81]]. In NSCLC patients with bone metastases, denosumab combined with ICIs leads to a significant improvement of OS and PFS [82,83], higher ORR [83], and no increase in immune-related adverse events [83]. Similarly, adding zoledronate to anti-PD-1 therapy improves OS of NSCLC patients with bone metastases [84].The combined effects of ICI and denosumab was also assessed at the biological level in the BONEMET study (melanoma with bone metastasis), showing increased CXCL-13 and IFN-γ levels, and enhanced CD8+ T cell responses with dual therapy [81]. Ongoing trials in NSCLC, melanoma, and RCC are investigating this combination therapy further (Table 2).

**Table 2:** Clinical trials involving patients with cancer and bone metastases treated with concomitant immune checkpoint inhibitor and denosumab therapy.

Clinical trials involving patients with cancer and bone metastases treated with concomitant ICI and denosumab therapy (*)								
Cancer type	ICI Treatment (⁎⁎)	Study type	Total number of patients (n)	ICI (n)	ICI+denosumab (n)	Outcome of patients treated with ICI vs ICI+ denosumab	P value	Reference
**Melanoma**	Nivolumab, Pembrolizumab or Ipilimumab	retrospective	37	26	11	OS; 22.8 vs 57 months	0.48	Afzal, 2018[75]
	Ipilimumab + Nivolumab	retrospective	13	0	13	ORR; 54% (ICI+ denosumab)	NA	Angela, 2019[76]
	Nivolumab		16	0	16	ORR; 50% (ICI+ denosumab)	NA	
	Nivolumab, Pembolizumab or Ipilimumab	retrospective	66	0	66	OS; 8.4 vs 28.9 weeks (#)	0.44	Liede, 2018[77]
	Ipilimumab and Nivolumab	retrospective	34	16	18	PFS; 4.4 vs 2.9 months	NR	Schaper, 2024[78]
**NSCLC**	Pembrolizumab or Nivolumab	retrospective	241	0	241	OS; 8.3 vs 16.6 weeks*	<0.0001	Liede, 2018[77]
	Pembrolizumab, Nivolumab, Atezolizumab or Durvalumab	retrospective	86	39	47	OS; 14.2 vs 8.6 months	0.02	Asano, 2024[79]
	Pembrolizumab, Nivolumab, Atezolizumab or Ipilimumab	retrospective	69	0	69	OS; 3.6 vs 11.5 months ($)	0.0005	Cao, 2021[80]
	Pembrolizumab, Nivolumab or Atezolizumab	retrospective	24	16	6	PFS; 4.2 vs 15.9 months	<0.001	Bongiovanni, 2021[82]

(*) ICI = immune check point inhibitor; NA = not applicable; NR = not reached; NSCLC = non-small cell lung cancer; OS = overall survival; ORR = objective response rate; PFS = progression free survival.

(**) ICIs: anti-PD-1 (Pembrolizumab, Nivolumab), anti-PDL-1 (Atezolizumab, Durvalumab), and anti-CTLA-4 (Ipilimumab) checkpoint inhibitors.

(#) comparison of short treatment period of ICI+ denosumab (<6 weeks) vs longer period (>6 weeks).

($) comparison of short treatment period of ICI+ denosumab (<3 months) vs longer period (>3 months).

Combined ICI therapies comparing denosumab and zoledronate have only been conducted in patients with advanced NSCLC [85,86]. Retrospective data from the Italian Bone Metastasis Register in NSCLC patients showed that bone-targeted therapy (denosumab or zoledronate) improved OS when combined with ICI therapy [85]. In this retrospective study, denosumab tends to improve PFS more efficiently and more rapidly than zoledronate (PFS 15.9 months; 95 %CI, 5.1–not estimable, p=0.068). Similarly, denosumab outperforms zoledronate in ICI-treated NSCLC patients with high bone tumor burden (more than 3 skeletal lesions) [PFS: 15.2 (95 % CI 0.1–30.7) vs 5.4 months (95 % CI 0.1–10.7, p=0.002] [86].

Overall, these experimental and clinical findings suggest that blocking osteoclast-mediated bone resorption with denosumab or bisphosphonates could potentially improve extraosseous tumor response to ICI therapy in patients with cancer and bone metastases. Furthermore, denosumab could offer added immunomodulatory benefits beyond bone protection.

## Conclusion and perspectives

The interplay between cancer cells, bone cells, and immune cells in the bone marrow gives rise to an immunosuppressive environment that helps disseminated tumor cells to hide from cancer immunosurveillance (Fig. 1). This “cold” immune environment poses significant challenges in the clinical management of metastatic bone disease, as ICI therapies shorten the survival of patients with cancer and bone metastases, compared to ICI-treated cancer patients without bone metastases (Table 1). However, bone-targeted therapies, such as bisphosphonates and denosumab, are a valuable treatment approach not only to mitigate skeletal complications in patients with bone metastases, but also improve clinical outcomes of patients with bone metastases treated with ICIs. In addition, combining denosumab with ICIs enhances the immune response in patients with bone metastases (Table 2). Furthermore, both experimental and clinical evidence suggest that initiating ICI therapy prior to denosumab significantly improves outcomes [87,88]. Specifically, in a mouse model of subcutaneous colon cancer (CT26), Ahern *et al.* demonstrated that ICI treatment followed by denosumab markedly reduced tumor growth compared to ICI therapy alone, whereas administrating denosumab before ICI had the opposite effect [87]. Similarly, in a retrospective registry of patients with bone-metastatic NSCLC mainly, Mabrut *et al.* reported that the sequence of ICI followed by denosumab was associated with improved treatment outcomes [88]. These preclinical and clinical findings strongly suggest that patients who had already been exposed to denosumab should not be eligible for receiving ICI therapy. Future research should therefore focus on optimizing this combination therapy to improve clinical outcomes of cancer patients with bone metastases and to extend these clinical trials to patients with advanced cancer at high risk for bone relapse. Furthermore, irrespective of the presence or absence of bone metastases, patients with cancer who are treated with ICIs have an increased fracture risk, suggesting a negative impact of ICI therapy on bone homeostasis. Thus, a better understanding of the effect of ICI therapies on specific immune interactions within the bone marrow microenvironment is necessary to enable the development of more effective and personalized therapeutic strategies.

## Data sharing

Data sharing is not applicable to this article as no new data were created or analyzed in this study.

## CRediT authorship contribution statement

**E. Massy:** Writing – review & editing, Writing – original draft, Visualization, Validation, Methodology, Conceptualization. **C.B. Confavreux:** Writing – review & editing, Validation, Conceptualization. **M. Point:** Writing – review & editing, Validation. **E. Bonnelye:** Writing – review & editing, Validation, Conceptualization. **P. Clézardin:** Writing – review & editing, Validation, Formal analysis, Conceptualization.

## Declaration of competing interest

In accordance with Taylor & Francis policy and ethical obligation as a researcher, CC reports he gave talks for Amgen Inc, BMS and MSD and received research grants from Amgen Inc and MSD. EM, EB and PC report no conflict of interests.

## Appendix Supplementary materials

Image, application 1
